# A newly identified homologous SxtPR efflux system contributes to sulfamethoxazole/trimethoprim susceptibility and pathogenesis in *Klebsiella pneumoniae*

**DOI:** 10.1128/aac.01644-25

**Published:** 2026-06-18

**Authors:** Jordi Corral, María Pérez-Varela, Marc Gaona, Susana Campoy, Jordi Barbé, Jesús Aranda

**Affiliations:** 1 Departament de Genètica i de Microbiologia, Facultat de Biociènces, Universitat Autònoma de Barcelona 16719, Barcelona, Spain; Universita degli Studi di Roma La Sapienza, Rome, Italy

**Keywords:** *Klebsiella pneumoniae*, MFS efflux pump, LysR−type transcriptional regulator

## Abstract

*Klebsiella pneumoniae* is an opportunistic pathogen responsible for a wide range of healthcare-associated infections. In a recent work, we identified a novel Major Facilitator Superfamily efflux system in *Acinetobacter baumannii*, designated SxtPR, which mediates antimicrobial resistance and pathogenicity (M. Gaona, J. Corral, S. Campoy, J. Barbé, et al, Antimicrob Agents Chemother e0071224, 2024, https://doi.org/10.1128/aac.00712-24). Here, we report the identification and characterization of a homologous system in *Klebsiella pneumoniae* that plays a similar dual role in antimicrobial resistance and virulence.

## INTRODUCTION

*Klebsiella pneumoniae* is a gram-negative, encapsulated opportunistic pathogen that primarily colonizes the human oropharynx and gastrointestinal tract (1). It is a major cause of healthcare-associated infections, including pneumonia, urinary tract infections, bloodstream infections, meningitis, and device-associated infections, particularly in immunocompromised patients (2). The emergence of multidrug-resistant and hypervirulent strains of *K. pneumoniae* has raised significant public health concerns due to their resistance to multiple antibacterial agents (2–4).

In bacteria, antimicrobial resistance mechanisms include drug inactivation, target modification, altered permeability, and active efflux (4, 5). Efflux transport systems extrude not only antimicrobials and toxic compounds but also molecules involved in biofilm formation and virulence (5–7). In *K. pneumoniae*, AcrAB, OqxAB, EefAB, and KexD efflux systems are associated with antimicrobial resistance and pathogenicity (8, 9). Recently, we identified the SxtP transporter in *Acinetobacter baumannii*, a Major Facilitator Superfamily (MFS) efflux pump that contributes to trimethoprim/sulfamethoxazole (SXT) resistance, surface-associated motility, and virulence (10). Additionally, this efflux pump appears to be regulated by the LysR-type transcriptional activator SxtR, whose inactivation produces phenotypic effects similar to those observed in the SxtP-deficient strain (10). In the present study, we described a SxtPR homologous system in *K. pneumoniae*.

**Fig 1:**
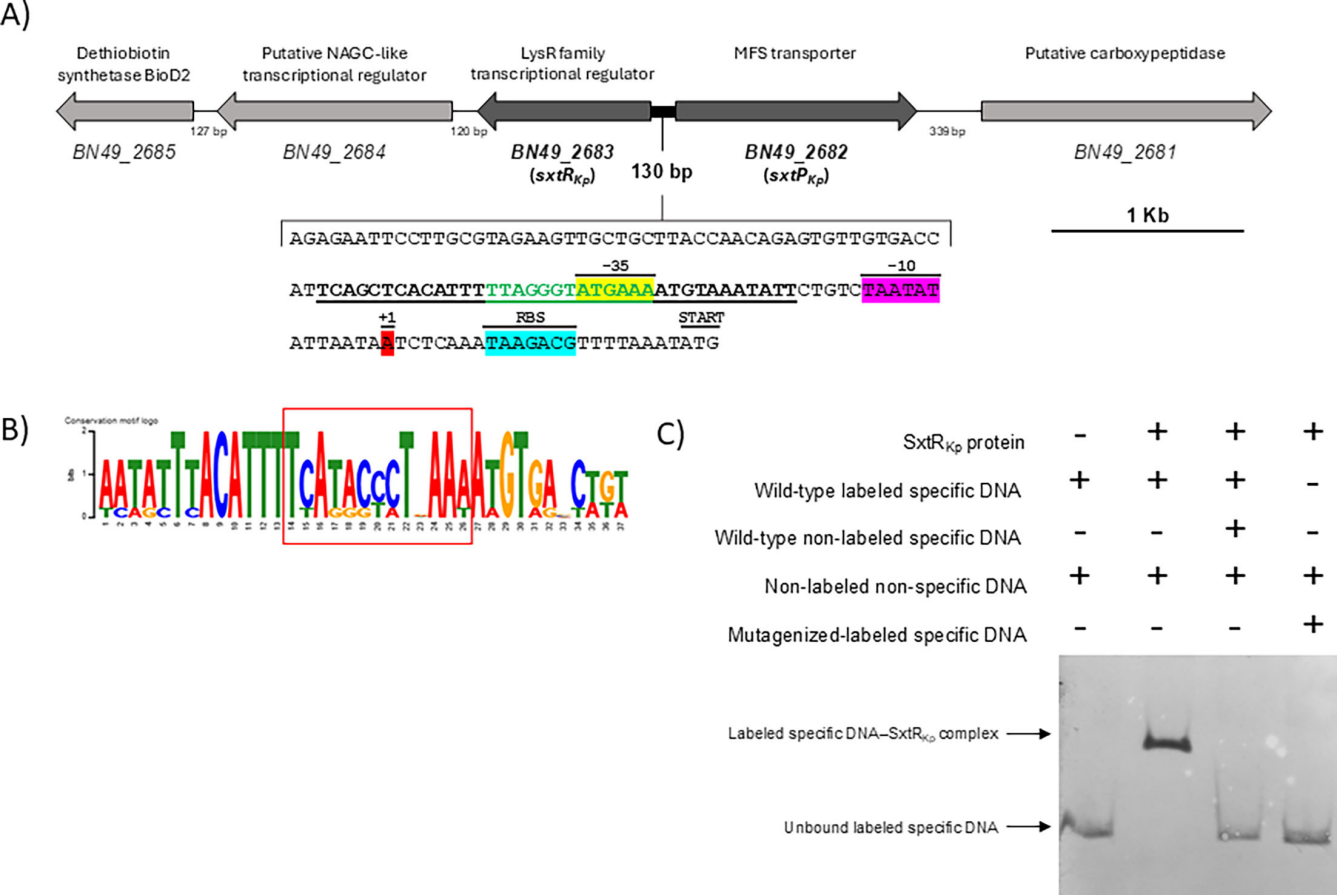
(**A**) Schematic representation of the intergenic region between the *BN49_2682* (*sxtP_Kp_*), *BN49_2683* (*sxtR_Kp_*), and surrounding genes in *K. pneumoniae* strain Kp52.145, highlighting the 130-bp intergenic sequence. Strain Kp52.145, highlighting the 130-bp intergenic sequence. A putative LysR motif (T−N₁₁−A) is indicated in green within the consensus sequence identified in panel B and is shown in bold and underlined. The −35 and −10 promoter regions, predicted using BPROM (Softberry), are highlighted in yellow and pink, respectively. The +1 transcription start site and the ribosome binding site (RBS), identified using the Salis Lab tools (https://salislab.net/software/), are indicated in red and blue, respectively. The start codon (ATG) of the MFS transporter is located at the end of the sequence. (**B**) Consensus sequence generated using the Snowprint tool with regulator BN49_2683 (GenBank CDO14439.1) as the query sequence; resulting sequences are shown in Table S3. (**C**) Competitive electrophoretic mobility shift assay (EMSA) of purified SxtR_Kp_ protein with a digoxigenin-labeled DNA fragment corresponding to the intergenic region between *BN49_2682* (*sxtP_Kp_*) and *BN49_2683* (*sxtR_Kp_*). The unlabeled specific DNA corresponds to the same intergenic region without digoxigenin and was added in a 10-fold molar excess as a non-labeled competitor. Salmon sperm DNA was added as non-specific DNA in all binding reactions. For each reaction, the components added (+) or omitted (−) from the binding mixture are indicated. All EMSAs were performed at least three times, and a representative image is shown. The mutagenized probe DNA is identical to the wild-type sequence except that the sequence TTAGGGTATGAAA was replaced with CCCAAACCCACCC.

To identify the homolog *A. baumannii* SxtPR system in *K. pneumoniae*, we screened the genome of strain Kp52.145 (GenBank FO834906.1) using the *sxtP* and *sxtR* genes of *A. baumannii* ATCC 17978 as reference (GenBank APP30283.1 and APP30282.1, respectively). This search identified two genes in *K. pneumoniae: BN49_2682* (CDO14438.1) and *BN49_2683* (CDO14439.1), predicted to encode an MFS transporter, and a LysR-type regulator, respectively. These genes are divergently oriented and separated by a 130-bp intergenic region (Fig. 1A), closely resembling the *A. baumannii* SxtPR system (SxtPR_Ab_) (10). BN49_2682 (renamed as SxtP_Kp_) shares 41.8% identity and 97% coverage with SxtP_Ab_, whereas BN49_2683 (renamed as SxtR_Kp_) shares 37.1% identity and 92% coverage with SxtR_Ab_. Remarkably, a genome-wide analysis of 1,439 *K. pneumoniae* genomes, including clinical isolates, revealed that both genes are present in 84.1% of the strains, underscoring their broad conservation within the species (Table S1).

*K. pneumoniae* knockout strains lacking either the *sxtP_Kp_* or *sxtR_Kp_* gene were generated by in-frame deletion as previously described (11), using the oligonucleotides listed in Table S2. Antimicrobial susceptibility of the wild-type (WT) strain and knockout derivatives was determined by broth microdilution following Clinical and Laboratory Standards Institute guidelines (12). As shown in Table 1, deletion of either *sxtP_Kp_* or *sxtR_Kp_* markedly increased susceptibility to trimethoprim, sulfamethoxazole, co-trimoxazole (sulfamethoxazole/trimethoprim [SXT] 1:5). To confirm that antimicrobial susceptibility phenotypes were due to the lack of the SxtPR_Kp_ system, mutant strains were complemented by reintroducing the corresponding WT gene using the multicopy pBAV1Gm-T5-gfp plasmid (13). Restoration of the parental phenotype was confirmed by MIC values (Table 1), demonstrating that the observed phenotype is specifically attributable to the SxtP_Kp_ efflux pump and its LysR-type SxtR_Kp_ regulator. This phenotype resembles that of the *A. baumannii* lacking the *sxtP_Ab_* or *sxtR_Ab_* genes, although the SxtPR_Ab_ system extrudes a wide range of antimicrobials (10). No differences in susceptibility to other antimicrobials were observed in the *sxtP_Kp_ or sxtR_Kp_* knockouts, except for cefotaxime, while susceptibility to ceftazidime remained unchanged (Table 1). The increased cefotaxime susceptibility despite retained ceftazidime resistance resembles a dissociated phenotype previously reported in Enterobacterales. Differences in susceptibility between structurally related cephalosporins have been linked to differential β-lactamase activity (14) and, in some cases, to changes in RND efflux systems that selectively modulate cephalosporin susceptibility (15). In gram-negative bacteria, MFS transporters export compounds from the cytosol into the periplasm, which already reduces intracellular antibiotic levels. Although cooperation with tripartite RND systems can enhance efflux, it is not strictly required for this initial detoxification step. For example, in *A. baumannii*, TetA exports tigecycline into the periplasm, while RND pumps such as AdeABC and AdeIJK can further decrease susceptibility (16, 17).

**TABLE 1:** Minimum inhibitory concentrations (MICs, mg/L) of the indicated antimicrobials for *K. pneumoniae* strain Kp52.145 WT and its derivative mutants^*a*^

Antimicrobial	Kp52.145 (WT)	BN49_2683 (*sxtR_Kp_*)	BN49_2683 (*sxtR_Kp_*) complemented	BN49_2682 (*sxtP_Kp_*)	BN49_2682 (*sxtP_Kp_*) complemented
Trimethoprim	4	**0.32**	4	**0.32**	4
Sulfamethoxazole	256	**2**	256	**2**	256
Co-trimoxazole	128	**2**	128	**2**	128
Cefotaxime	8	**0.32**	8	**0.32**	8
Ceftazidime	0.5	0.5	0.5	0.5	0.5
Ampicillin	256	256	256	256	256
Ticarcillin	8	8	8	8	8
Ciprofloxacin	0.032	0.032	0.032	0.032	0.032
Levofloxacin	0.032	0.032	0.032	0.032	0.032
Rifampin	32	32	32	32	32
Minocycline	4	4	4	4	4
Tetracycline	4	4	4	4	4
Amikacin	2	2	2	2	2
Gentamicin	0.5	0.5	0.5	0.5	0.5
Apramycin	4	4	4	4	4
Erythromycin	128	128	128	128	128
Chloramphenicol	8	8	8	8	8
Colistin	4	4	4	4	4

^*a*^ Antimicrobials with changes in MIC are indicated in bold.

The fact that both *sxtP_Kp_* and *sxtR_Kp_* knockout strains display a similar antimicrobial susceptibility profile (Table 1) suggests that the regulator acts as an activator, as reported for the SxtPR_Ab_ system (10). To confirm this, we performed reverse transcription quantitative PCR analyses to evaluate the transcript levels of the *sxtP_Kp_* gene in the *sxtR_Kp_* knockout mutant and compared to the WT strain. The expression levels of the MFS efflux pump gene were normalized to the transcription level of the 16S ribosomal RNA housekeeping gene using the threshold cycle (2^−ΔΔCT^) method (18). The results showed twofold reduction in gene expression (Fig. S1). To further investigate the regulatory interaction, we performed *in silico* analysis using the SxtR_Kp_ regulator BN49_2683 (GenBank: CDO14439.1) as the query sequence with the Snowprint tool (https://snowprint.groov.bio/) (19). This analysis identified a highly conserved sequence (Table S3) that is specifically present upstream of the *sxtP_Kp_* ORF (Fig. 1B), which contains a T–N₁₁–A motif characteristic of LysR-type transcriptional regulators (20). Notably, this DNA motif is located 54-bp upstream of the SxtR_Kp_ start codon, the same distance as the corresponding LysR box in *A. baumannii* (10). Afterward, electrophoretic mobility shift assay (EMSA) experiments, performed as previously described (10), demonstrated that purified SxtR_Kp_ protein specifically binds to the 130-bp intergenic region, supporting its role as a direct regulator, similar to SxtR_Ab_ in *A. baumannii*. Finally, synthetic 100-bp oligonucleotides encompassing either the native putative binding region or a mutated version confirmed that the identified sequence is involved in the SxtR_Kp_ binding to the MFS promoter (Fig. 1C).

**Fig 2:**
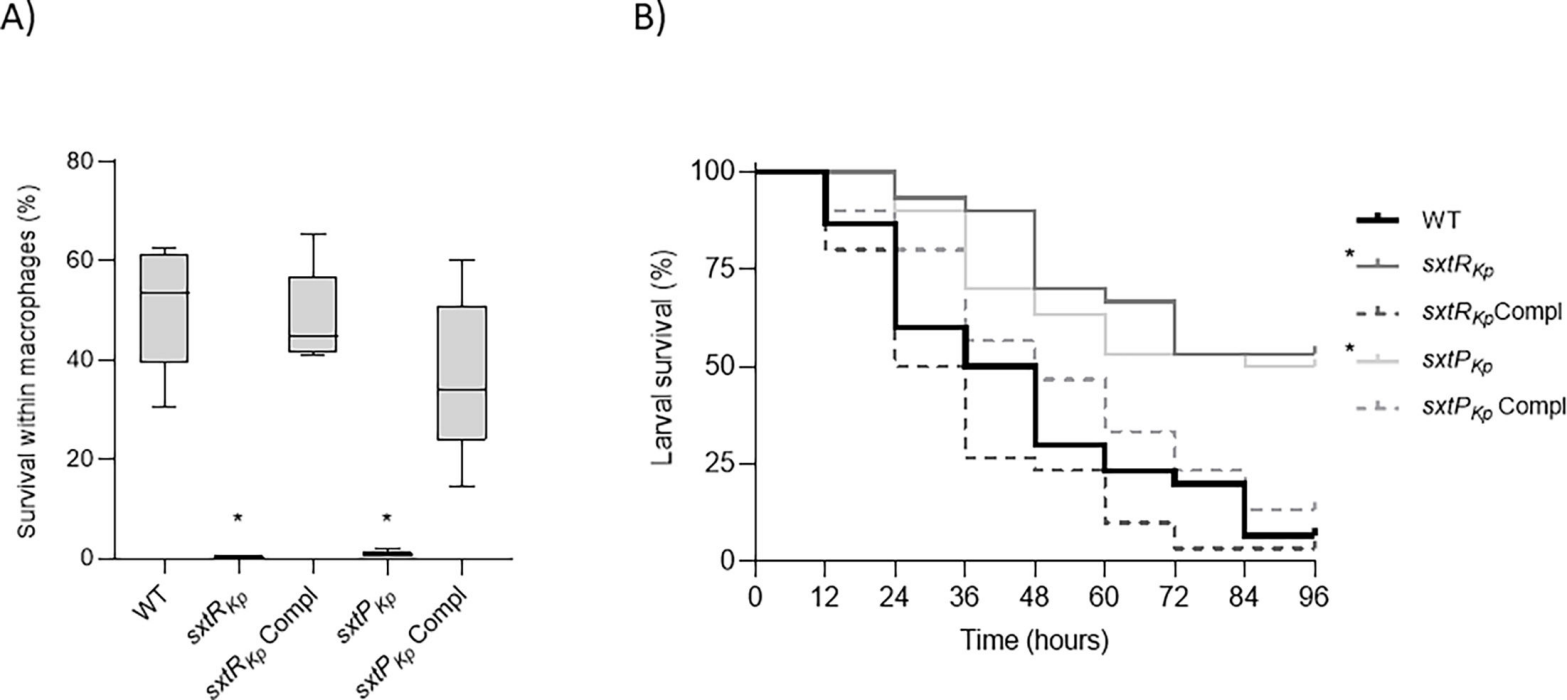
(**A**) Relative intracellular survival of the indicated *K. pneumoniae* strains within J774A.1 murine macrophages. Values represent the average CFU recovered after infection, normalized to the initial bacterial inoculum. Error bars indicate standard deviation from four replicates across three independent biological experiments. Data were analyzed using a two-tailed, one-way analysis of variance followed by Tukey’s *post hoc* test for multiple comparisons. Asterisks denote statistical significance (*P < 0.001). (**B**) *G. mellonella* killing assay of the specified strains. Larvae (*n* = 10 per group) were inoculated with ~10^5^ CFU of each strain or phosphate-buffered saline as a negative control (not shown). *P < 0.001 compared to the parental (WT) strain. Survival curves were plotted using the Kaplan-Meier method, and differences in survival were evaluated using the log-rank test. All experiments were independently repeated at least three times, and a representative survival curve is shown.

Efflux pumps are traditionally associated with antimicrobial resistance, but increasing evidence links them to pathogenic processes, including immune evasion and host colonization (5, 6, 21). In *K. pneumoniae*, several efflux systems such as AcrAB, OqxAB, EefAB, and KexD have been reported to protect against harmful compounds, thereby facilitating colonization and virulence (8, 9, 22). While several efflux pumps have been linked to motility and pathogenesis in *A. baumannii* (21), *K. pneumoniae* is a non-motile bacterium (23), prompting us to investigate whether the newly identified SxtPR_Kp_ efflux system contributes to virulence. To address this, we evaluated the survival of knockout mutants lacking either the LysR-type regulator (*sxtR_Kp_*) or the MFS efflux pump (*sxtP_Kp_*) within J774A.1 murine macrophages, as previously described (24, 25). The *sxtR_Kp_* knockout exhibited a significant reduction in intracellular survival compared to the WT strain (Fig. 2A). Similarly, the *sxtP_Kp_* knockout also showed reduced survival (Fig. 2A), supporting the involvement of both genes in *K. pneumoniae* pathogenesis. Virulence was further evaluated using the *Galleria mellonella* infection model (26). Larvae infected with either *sxtR_Kp_* or *sxtP_Kp_* knockout mutants showed significantly higher survival rates than those infected with the WT strain (Fig. 2B), indicating that the SxtPR_Kp_ system substantially contributes to *K. pneumoniae* pathogenesis during host infection. Additionally, as observed in the MIC determinations, complemented knockout mutants restored parental phenotype in both macrophage infection and *G. mellonella* virulence experiments (Fig. 2), demonstrating that the observed phenotypes are specifically attributable to the SxtPR_Kp_ system.

The involvement of efflux pumps in virulence has been widely documented in multidrug-resistant pathogens. In *Escherichia coli*, the AcrAB-TolC efflux pump contributes to both antibiotic resistance and host colonization (27); while in *Shigella flexneri*, the MFS efflux pump EmrKY is essential for survival within macrophages (28). Similarly, the MacAB ABC-type pump of *Salmonella enterica* is required for preventing oxidative stress during intracellular survival (29). In *K. pneumoniae*, the AcrAB efflux system has been linked to bile salt resistance and virulence, as mutants of the *acrA* and *acrB* genes show reduced pathogenicity in *G. mellonella* (30) and decreased resistance to innate immune defenses (22). Additionally, overexpression of the OqxAB pump, caused by mutations in the *oqxR* transcriptional repressor, has been linked to enhanced antimicrobial resistance and increased virulence, especially when co-expressed with AcrAB, as shown in the *Caenorhabditis elegans* model (31). Based on these observations, we hypothesize that in *K. pneumoniae* the SxtPR system could contribute to virulence either by extruding toxic compounds that protect the bacterium or by exporting virulence factors that directly promote pathogenicity.

In conclusion, our work identifies the SxtPR_Kp_ system as contributing to *K. pneumoniae* pathogenicity. Following our recent characterization of the homologous system in *A. baumannii* (10), we demonstrate that the MFS pump SxtP_Kp_ and its LysR-type activator SxtR_Kp_ are also widely conserved among *K. pneumoniae* strains, including clinical isolates. Both *K. pneumoniae* and *A. baumannii* are major nosocomial pathogens responsible for healthcare-associated infections, with this system contributing to antimicrobial resistance, particularly against co-trimoxazole. Our findings highlight the SxtPR system as a conserved mechanism of antimicrobial resistance and virulence in critical-priority bacterial pathogens, underscoring its potential as a novel therapeutic target against multidrug-resistant nosocomial pathogens.

## SUPPLEMENTAL MATERIAL

Fig. S1 aac.01644-25-s0001.tif RT-qPCR analyses to evaluate the transcript levels of the sxtPKp gene in the sxtRKp knockout mutant and compared to the WT strain.

Table S1 aac.01644-25-s0002.xlsx *In silico* genomic analysis of sxtRKp and sxtPKp.

Table S2 aac.01644-25-s0003.docx Oligonucleotides used in this work.

Table S3 aac.01644-25-s0004.docx Predicted DNA-binding motif of the SxtRKp regulator.

ASM does not own the copyrights to Supplemental Material that may be linked to, or accessed through, an article. The authors have granted ASM a non-exclusive, world-wide license to publish the Supplemental Material files. Please contact the corresponding author directly for reuse.
