# Supplementary material for: A newly identified homologous SxtPR efflux system contributes to sulfamethoxazole/trimethoprim susceptibility and pathogenesis in Klebsiella pneumoniae
Source: Antimicrob Agents Chemother. 2026 Jun 18;70(8):e01644-25. doi: 10.1128/aac.01644-25 (PMC13436341; doi:10.1128/aac.01644-25)
Supplement: Supplementary file 1 [file aac.01644-25-s0003.docx]

**Supplementary Table 2. Oligonucleotides used in this work.**

| Name | Sequence (5’ → 3’) | Application |
| --- | --- | --- |
| pUA1108-2683_Fw | actgCATATGAATATCGAGTTGCGTC | Cloning for StxR_Kp_ protein overexpression |
| pUA1108-2683-His_Rv | actgGGATCCTTA**ATGATGATGATGATGATG**gccgccgccATTATGCTCGCTTTTCCAGG | Cloning for StxR_Kp_ protein overexpression |
| -108pUA1108 | CCGACATCATAACGGTTC | Sequencing primer for pUA1108 vector |
| +128pUA1108 | AGACAAGCTGTGACCGTC | Sequencing primer for pUA1108 vector |
| p2682_Fw | AGAGAATTCCTTGCGTAGAAG | Cloning for intergenic region amplification |
| p2682_Rv | ATTTAAAACGTCTTATTTGAGATT | Cloning for intergenic region amplification |
| p2682_WT_Fw | TGCTGCTTACCAACAGAGTGTTGTGACCATTCAGCTCACATTTTTAGGGTATGAAAATGTAAATATTCTGTCTAATATATTAATAATCTCAAATAAGACa | Synthetic oligo to obtain the WT *stxP_Kp_* promoter EMSA probe |
| p2682_WT_Rv | GTCTTATTTGAGATTATTAATATATTAGACAGAATATTTACATTTTCATACCCTAAAAATGTGAGCTGAATGGTCACAACACTCTGTTGGTAAGCAGCAa | Synthetic oligo to obtain the WT *stxP_Kp_* promoter EMSA probe |
| p2682_Mut_Fw | TGCTGCTTACCAACAGAGTGTTGTGACCATTCAGCTCACATTTcccaaacccacccATGTAAATATTCTGTCTAATATATTAATAATCTCAAATAAGACa | Synthetic oligo to obtain the mutant *stxP_Kp_* promoter EMSA probe |
| p2682_Mut_Rv | GTCTTATTTGAGATTATTAATATATTAGACAGAATATTTACATgggtgggtttgggAAATGTGAGCTGAATGGTCACAACACTCTGTTGGTAAGCAGCAa | Synthetic oligo to obtain the mutant *stxP_Kp_* promoter EMSA probe |
| 16S_Fw | GATGCAACGCGAAGAACC | RT-qPCR assays |
| 16S_Rv | CGTAAGGGCCATGATGACTT | RT-qPCR assays |
| 2682-RT-qPCR_Fw | ATCAGCAGAAAACCTTCGAT | RT-qPCR assays |
| 2682-RT-qPCR_Rv | GGGGTCTTTACCGATTTCTT | RT-qPCR assays |
| M13FpUC | *GTTTTCCCAGTCACGAC | Sequencing primer for pGEMT vector |
| M13RpUC | *CAGGAAACAGCTATGAC | Sequencing primer for pGEMT vector |
| Mut-2683_Fw | CAGCTCACATTTTTAGGGTATGAAAATGTAAATATTCTGTCTAATATATTAATAATCTCAAATAAGACGTTTTAAATGCGGTTTTATGGACAGCAAGC | *stxR_Kp_* mutant construction by lambda Red recombination |
| Mut-2683_Rv | GTGAAGCACTTCGACGGTGAACGTCAAATTTTTACTTAGCCTTGTGATTTACCGCACATTTCCAGCCCCTTTTCGCCTCAGAAGAACTCGTCAAGAAGGC | *stxR_Kp_* mutant construction by lambda Red recombination |
| Com-2683_Fw | CCAACAGAGTGTTGTGACC | *stxR_Kp_* mutant verification |
| Com-2683_Rv | CCCTTATTTTCGCGCTTCG | *stxR_Kp_* mutant verification |
| Compl-2683_Fw | gcatTCTAGAAGAGAATTCCTTGCGTAGAAG | *stxR_Kp_* mutant complementation |
| Compl-2683_Rv | gcatTCTAGATCAATTATGCTCGCTTTTCC | *stxR_Kp_* mutant complementation |
| Mut-2682_Fw | GATGGGCTTGTCTTCCATAATGGGCATGTGGTGAAACGTGGCCCGCAGGCCACGTCACACGCGACTATTTCAGTCGGCGGTTTTATGGACAGCAAGC | *stxP_Kp_* mutant construction by lambda Red recombination |
| Mut-2682_Rv | GAGCTGAATGGTCACAACACTCTGTTGGTAAGCAGCAACTTCTACGCAAGGAATTCTCTGTGAGTCGTACAACAACCTCAGAAGAACTCGTCAAGAAGGC | *stxP_Kp_* mutant construction by lambda Red recombination |
| Com-2682_Fw | GTTTATTGCACTGCTGTCCG | *stxP_Kp_* mutant verification |
| Com-2682_Rv | GTAGCGCAAATGACGCAACTCG | *stxP_Kp_* mutant verification |
| Compl-2682_Fw | gcatTCTAGAGTGAGTCGTACAACAACCGTTG | *stxP_Kp_* mutant complementation |
| Compl-2682_Rv | gcatTCTAGACTATTTCAGTCGCTGATGTAAGC | *stxP_Kp_* mutant complementation |
| pBAV_Fw | CACTGTTCCTTGCATTCTA | Sequencing primer for pBAV1Gm-T5-gfp |
| pBAV_Rv | ATTGGGACAACTCCAGTGAA | Sequencing primer for pBAV1Gm-T5-gfp |

Sequences recognized by restriction endonuclease sites are underlined (CATATG: *Nde*I, GGATCC: *BamH*I and TCTAGA: *Xba*I), 6×His tags are indicated in bold, and Gly spacers and restriction site tails in lower case.

* When required, primers were 5′-labeled with DIG for EMSA experiments.
