# Supplementary material for: A newly identified homologous SxtPR efflux system contributes to sulfamethoxazole/trimethoprim susceptibility and pathogenesis in Klebsiella pneumoniae
Source: Antimicrob Agents Chemother. 2026 Jun 18;70(8):e01644-25. doi: 10.1128/aac.01644-25 (PMC13436341; doi:10.1128/aac.01644-25)
Supplement: Supplementary file 2 [file aac.01644-25-s0004.docx]

**Supplementary Table 3. Predicted DNA-binding motif of the SxtR_Kp_ regulator.**

| Uniprot ID | Identity | Coverage | Genome | Start | Stop | Strand | Predicted operator | Align score |
| --- | --- | --- | --- | --- | --- | --- | --- | --- |
| A0A0C7KBQ4 | 99.7 | 100 | NC_009648.1 | 1749841 | 1750743 | + | tcagtCCACATTTTTAGGGTATGAAAATGTGAatattt | 46.5 |
| A0A48DP1D2 | 99 | 100 | CAAHCU01000001.1 | 1756196 | 1756098 | - | aaatTTACATTTTTCATACCCTAAAAATGTGAgctga | 31.5 |
| A0A0B7GB32 | 97.3 | 100 | CAJ0XS02000001.1 | 1104290 | 1105192 | - | aaatTTACATTTTTCATACCCTAAAAATGTGAgctga | 31.5 |
| A0A0H3WJV3 | 88.4 | 98 | CP003218.1 | 4652308 | 4653201 | - | aaatTTACATTTTTCATACCCTAAAAATGTGAccatt | 27.5 |
| A0A1Y6GMG9 | 87.4 | 98 | NKYI01000022.1 | 35501 | 36403 | - | aaatTTACATTTTTCATACCCTAAAAATGTGAtctgt | 31.5 |
| A0A1V2BKA3 | 85.3 | 100 | CAADJG01000002.1 | 2872691 | 2873593 | - | aaatTTACATTTTTCATACCTAAAAATGTGAcctgt | 31.5 |

Predicted DNA-binding sequence of the SxtR_Kp_ regulator based on the Snowprint tool (<https://snowprint.groov.bio/>). BLAST results were filtered using identity and coverage cutoffs of 85% and 90%, respectively.
